# A Conformational Sampling Model for Radical Catalysis in Pyridoxal Phosphate- and Cobalamin-dependent Enzymes*

**DOI:** 10.1074/jbc.M114.590471

**Published:** 2014-09-11

**Authors:** Binuraj R. K. Menon, Karl Fisher, Stephen E. J. Rigby, Nigel S. Scrutton, David Leys

**Affiliations:** From the Manchester Institute of Biotechnology, Faculty of Life Sciences, The University of Manchester, Manchester M1 7DN, United Kingdom

**Keywords:** Adenosylcobalamin (AdoCbl), Electron Paramagnetic Resonance (EPR), Protein Dynamic, Pyridoxal Phosphate, Radical, Conformational Sampling, Domain Dynamics, Ornithine Aminomutase

## Abstract

Cobalamin-dependent enzymes enhance the rate of C–Co bond cleavage by up to ∼10^12^-fold to generate cob(II)alamin and a transient adenosyl radical. In the case of the pyridoxal 5′-phosphate (PLP) and cobalamin-dependent enzymes lysine 5,6-aminomutase and ornithine 4,5 aminomutase (OAM), it has been proposed that a large scale domain reorientation of the cobalamin-binding domain is linked to radical catalysis. Here, OAM variants were designed to perturb the interface between the cobalamin-binding domain and the PLP-binding TIM barrel domain. Steady-state and single turnover kinetic studies of these variants, combined with pulsed electron-electron double resonance measurements of spin-labeled OAM were used to provide direct evidence for a dynamic interface between the cobalamin and PLP-binding domains. Our data suggest that following ligand binding-induced cleavage of the Lys^629^-PLP covalent bond, dynamic motion of the cobalamin-binding domain leads to conformational sampling of the available space. This supports radical catalysis through transient formation of a catalytically competent active state. Crucially, it appears that the formation of the state containing both a substrate/product radical and Co(II) does not restrict cobalamin domain motion. A similar conformational sampling mechanism has been proposed to support rapid electron transfer in a number of dynamic redox systems.

## Teaser

**Background:** Catalysis in ornithine 4,5-aminomutase (OAM) may involve large scale domain dynamics.

**Results:** A conformational equilibrium is established following release of Lys^629^ from the PLP cofactor.

**Conclusion:** Substrate binding triggers conformational sampling of the cobalamin-binding domain to support catalysis.

**Significance:** A model for radical catalysis involving domain conformational sampling is established for pyridoxal phosphate/cobalamin-dependent enzymes.

## Introduction

Free radical chemistry in nature underpins key metabolic transformations, with the enzymes involved depending on cofactors such as adenosylcobalamin or *S*-adenosylmethionine to generate transient radical species (1–4). Uncontrolled radical propagation presents an inherent danger to these enzymes, leading to enzyme inactivation and cellular damage. Enzymes that use adenosylcobalamin enhance the rate of C–Co bond cleavage generating cob(II)alamin and a transient adenosyl radical by as much as 10^12^-fold (5, 6). Importantly, C–Co bond cleavage is limited in absence of substrate, a feat that is achieved through different means in the various adenosylcobalamin-dependent enzymes (7–10). In the case of the aminomutases lysine 5,6-aminomutase and ornithine 4,5-aminomutase (OAM)^3^ (forming class III of the adenosylcobalamin-dependent enzymes), a role for large scale domain reorientation linked to substrate binding has been proposed (11–13).

In addition to the cobalamin cofactor, these enzymes contain an additional pyridoxal 5′-phosphate (PLP) cofactor, bound within the substrate-binding triose phosphate isomerase (TIM)-like β/α barrel domain. The PLP cofactor is covalently attached to a lysine residue (Lys^629^) that is derived from the cobalamin-binding Rossmann domain. Crucially, this covalent linkage can only occur when the Rossmann domain and associated cobalamin cofactor are located close to the *edge* of the TIM barrel. Other adenosylcobalamin-dependent enzymes such as glutamate mutase, ethanolamine ammonia lyase, or methylmalonyl-CoA mutase place the cobalamin-binding domain in close contact with the substrate-binding face of the TIM barrel (7, 9, 14, 15). Although structural changes induced by ligand binding are modest in glutamate mutase/ethanolamine ammonia lyase, methylmalonyl-CoA mutase uses a large scale conformational change (closure of the TIM barrel) to trigger catalysis by coupling the local structural changes induced by the binding of a relatively large methylmalonyl-CoA substrate (16, 17). These changes are required to form a catalytically competent active site.

**FIGURE 1.**
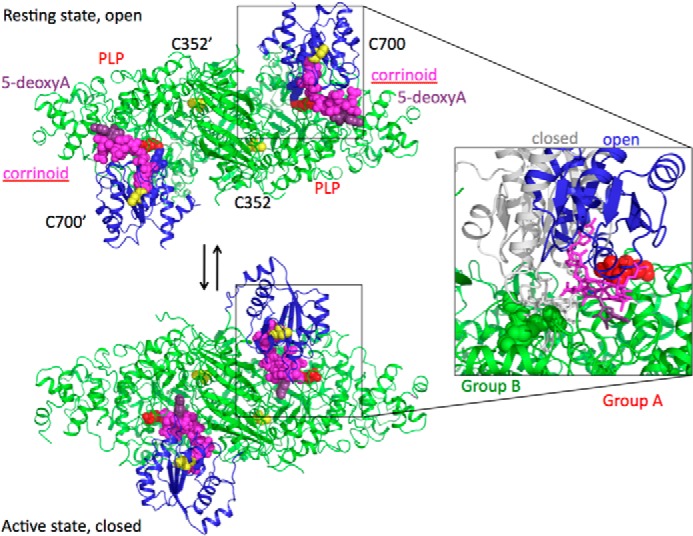
**Proposed conformations of OAM based on crystal structures and molecular simulations.** Open resting state and closed active state model for wild-type OAM. Wild-type cysteine residues are shown in *yellow spheres*, PLP cofactor is in *red spheres*, and adenosyl cobalamin is in *magenta spheres*. The *red balls* are used to indicate position of group A residues (Asp^627^ and Ile^424^), and *green balls* show group B residues (Glu^338^, Gly^339^, Pro^343^, and Gly^128^) in the enlarged figure section. The Rossmann domain is shown as *blue ribbon*. The open model is based on the published OAM crystal structure (Protein Data Bank code 3KP1), and the closed model is based on the model of Pang *et al.* (12).

A more dramatic change is proposed to occur for the PLP- and cobalamin-dependent aminomutases, where large scale domain motion follows substrate binding to the PLP, effectively moving from the edge to the center of the TIM barrel (11–13). This is facilitated by the severing of the covalent link between PLP and the cobalamin-binding domain (via Lys^629^) through formation of the substrate-PLP aldimine complex. Crystal structures are available for OAM soaked with substrate and inhibitors, revealing that ligand binding induces disorder in the cobalamin-binding domain (11). These structures were obtained under aerobic conditions resulting in oxidation of cob(II)alamin and displacement of the adenosine moiety. By contrast, under anaerobic conditions, transimination occurs in the crystal in the absence of domain reorientation. This suggests that the proposed equilibrium between the “open” (*i.e.* the nonactive state) and the “closed” (active state) states is poised toward the open state in the crystalline form of OAM. Unfortunately, no crystal structure is available for the aminomutase in the closed (active state), although plausible models have been proposed based on the similarities with glutamate mutase (see Fig. 1). It is interesting to note that large scale dynamic reorientation of cobalamin domains similar to what is proposed here has been directly observed in cobalamin-dependent methyltransferase complexes (18–20).

Here we have used MTSL spin-labeled OAM to study the distribution of distances between spin labels and between a spin label and the Co(II) radical using pulsed EPR methods (*i.e.* PELDOR). These studies reveal significant substrate dependent shifts in distances compatible with the proposed dynamic model for OAM catalysis. We used site-directed mutagenesis to further validate the proposed dynamic model for OAM catalysis by probing the role of key residues found at the Rossmann/TIM barrel domain interface in populating the open (inactive) and closed (active) conformations. Steady-state and single turnover kinetic studies of these variants are used to provide further evidence for a dynamic model for catalysis and to support a location for the docking site of the cobalamin-binding domain in close proximity to the PLP cofactor in the closed (active) form of the enzyme. Furthermore, EPR studies of freeze-quenched samples of wild-type OAM in presence of d-ornithine are consistent with the proposed multiple conformations of the OAM cobalamin domain. Our data suggest that, after substrate binding to OAM, the cobalamin-binding domain samples conformational space. It would appear that a strict choreography between domain motion and radical catalysis does not occur, *i.e.* the active Co(II) state is not restricting cobalamin domain motion. Such a mechanism is clearly more simple to implement on a molecular level, and thus likely more robust, and is akin to the sampling of the flavin domain in electron-transferring flavoprotein complexes (21–23).

## EXPERIMENTAL PROCEDURES

### 

#### 

##### Materials

AdoCbl, PLP, d-ornithine, dl-2,4-diaminobutyric acid (DABA), dl-2,3-diaminopropanioc acid (DAPA), trypsin, and glucose oxidase (from *Aspergillus niger*) were obtained from Sigma. *S*-(2,2,5,5-Tetramethyl-2,5-dihydro-1H-pyrrol-3-yl)methyl methanesulfonothioate (MTSL) was from Toronto Research Chemicals Inc. Rosetta(DE3)pLysS and other competent cells were purchased from EMD Biosciences.

##### Preparation of OAM Variants and Enzyme Purification

The single mutations were introduced into the pET-OAMH2 vector harboring the C-terminal hexahistidine-tagged OAM from *Clostridium sticklandii*, using QuikChange site-directed mutagenesis kit (Agilent Technologies) (24, 25). The following primers (MWG Eurofins) were used to generate single mutated variants: GLY128ASP forward primer, 5′-CT CCT CAA GGG ATT GAT GGA GTA CCT A-3′; GLY128ASP reverse primer, 5′-GT TAT AGG TAC TCC ATC AAT CCC TTG A-3′; GLU338ALA forward primer, 5′-ATT ACT CCT GAC GCG GGA AGA AAC GTT CC-3′; GLU338ALA reverse primer, 5′-GG AAC GTT TCT TCC CGC GTC AGG AGT AAT-3′; GLY339TRP forward primer, 5′-C CTG ACG AGT GGA GAA ACG TTC CTT GG-3′; GLY339TRP reverse primer, 5′-C AAG GAA CGT TTC TCC ACT CGT CAG GA-3′; PRO343TRP forward primer, 5′-GGA AGA AAC GTT TGG TGG CAT ATA TA-3′; PRO343TRP reverse primer, 5′-GTA TAT ATG CCA CCA AAC GTT TCT TCC-3′; ILE424GLU forward primer, 5′-GGA GAT GGA GAA GCA AGA CAA ATA AAT G-3′; ILE424GLU reverse primer, 5′-AT TTG TCT TGC TTC TCC ATC TCC ATT TC-3′; ASP627ALA forward primer, 5′-GA GAA GTA ATA GCT ATT AAA CAT GGC GG-3′; ASP627ALA reverse primer, 5′-ACC GCC ATG TTT AAT AGC TAT TAC TTC-3; CYS700SER forward primer, 5′-AAG ATT ATG ATC GGA TCC GGA GGA ACT CA-3′; and CYS700SER reverse primer, 5′-TG AGT TCC TCC GGA TCC GAT CAT AAT CTT-3′. The correct mutations were confirmed by complete plasmid DNA sequencing with MWG Eurofins (Covent Garden, UK).

The wild-type OAM and OAM variants were expressed and purified as described previously (24). The coupling enzyme (2*R*,4*S*)-2,4-diaminopentanoate dehydrogenase (DAPDH) was from *Clostridium difficile* with a C-terminal hexahistidine tag on DAPDH designated as pET-DAPDH vector (obtained as a gift from Dr. Kirsten Wolthers, University of British Columbia, Canada), which was expressed in a Rosetta(DE3)pLysS competent cells and purified as reported before (24).

##### Anaerobic Sample Preparation

In this study, holoenzymes (wild-type OAM, OAM variants, or DAPDH), reagent solution preparation, and anaerobic measurements were conducted in a Belle Technology anaerobic glove box (O_2_ levels < 1 ppm) under very dim light (to minimize photolysis of AdoCbl) unless otherwise stated. Buffer solution (100 mm NH_4_-EPPS, pH 8.5) was purged for 3 h with nitrogen and then brought into and allowed to equilibrate for 18 h in the glove box to prepare anaerobic buffer. Solid AdoCbl, PLP, d-ornithine, and inhibitors (DABA and DAPA) were introduced into the glove box and dissolved in anaerobic buffer. A concentrated protein sample was introduced into the glove box and gel-filtered using a 10-ml Econo-pack 10DG-desalting column (Bio-Rad) pre-equilibrated with anaerobic buffer. In preparing samples for protein labeling, 1 mm DTT (final concentration) was added to the concentrated enzyme to reduce the cysteine disulfides before passing through a desalting column. Excess oxygen was scavenged from eluted protein by glucose oxidase (13 units ml^−1^) and glucose (10 mm) to prepare anaerobic apoenzyme prior to use.

##### Anaerobic and Aerobic UV-visible Spectroscopic Measurements

Anaerobic UV-visible spectral changes of holo-OAM and variants upon binding with d-ornithine or DABA were followed in a Cary 50 UV-visible spectrophotometer (Varian Inc.) contained in an anaerobic glove box. The wild-type OAM or variant holoenzyme solutions contained 15 μm apoenzyme, 15 μm PLP, and 15 μm AdoCbl in a total volume of 1 ml in anaerobic buffer. Spectral changes for holo-OAM were recorded at 25 °C, at 0 s and 10 s and then at every 60 s up to 25 min following the addition of 2.5 mm d-ornithine or 2.5 mm DABA.

Aerobic UV-visible spectral changes of holo-OAM and variants upon binding with the inhibitor DAPA and subsequent changes on continuous photolysis were followed in a Cary 50 UV-visible spectrophotometer (Varian Inc.). The holoenzyme solution contained 30 μm apoenzyme (wild-type OAM or OAM variants), 30 μm PLP, and 30 μm AdoCbl in a total volume of 1 ml in aerobic buffer (100 mm NH_4_-EPPS, pH 8.5), and the reaction was initiated by adding 5 mm DAPA. After 25 min of incubation, holoenzyme was subjected to continuous illumination from a Schott KL1500 electronic light source, which provided illumination at an intensity of 1000 μmol m^−2^ s^−1^, and spectral changes were recorded at 25 °C at 0 and 10 s and then at every 60 s up to 25 min. A red insert filter (<530-nm cutoff filter) was attached to the light path to avoid protein photo-degradation.

##### Steady-state and Pre-steady-state Kinetic Measurements

Steady-state kinetic parameters for wild-type OAM and its variant forms were determined using an anaerobic coupled UV-visible spectrophotometric assay with DAPDH. Enzyme activity was measured by following absorbance increase at 340 nm, corresponding to reduction of NAD^+^ (Δϵ = 6220 m^−1^ cm^−1^) to NADH (24, 26). Reaction mixtures contained 100 nm holo-OAM, 100 nm DAPDH, 0.5 mm NAD^+^, and variable concentrations of d-ornithine (0–2500 μm) in anaerobic buffer. Steady-state assays were performed in a 1.0-ml total volume, 1-cm path length cuvette at 25 °C as previously described (24, 26).

Pre-steady-state kinetic measurements were performed in a stopped flow instrument (Applied Photophysics SX.17 MV) contained in an anaerobic glove box, essentially as described previously (25). Absorbance change at 528 nm was monitored for up to 0.25 s to follow AdoCbl Co-C bond homolysis by mixing holo-OAM or holo-OAM variants (25 μm after mixing) with d-ornithine (2.5 mm after mixing) or with DABA (2.5 mm after mixing) in anaerobic buffer at 25 °C (25). An average of 15–20 traces were averaged at each wavelength and used to fit to a single exponential equation to extract the observed rate constants.

##### Protein Labeling and Mass Spec Analysis of Labeled Cysteine

Highly concentrated anaerobic wild-type OAM holoenzyme (>600 μm) was incubated with five times molar excess of MTSL spin label for 4 h at room temperature in an anaerobic glove box. Excess spin label was then removed by size exclusion chromatography using a 10-ml Econo-pack 10DG desalting column, followed by dialysis using a 10-kDa molecular mass cutoff mini dialysis device (Thermo Scientific) against 2 liter of anaerobic buffer for 3 h. The dialyzed protein was then concentrated in a 10-kDa molecular mass cutoff Amicon ultra 0.5-ml centrifugal filter (Merck Chemicals) (27). The stoichiometry of cysteine labeling was verified by spin quantification EPR spectroscopy using standard MTSL solutions of known concentration. Samples for PELDOR measurements were prepared by mixing 250 μm labeled holoenzyme with 25 mm d-ornithine or with 25 mm DABA, and the samples were loaded into EPR tubes and frozen in liquid nitrogen within 3 min.

Determination of the site of MTSL labeling in OAM was achieved by protein digestion and mass spectrometry as follows. Labeled OAM protein (6 μl of 1 μg/μl stock concentration) was added to 45.8 μl of 25 mm ammonium bicarbonate followed by the addition of DTT (3 mm final concentration). Samples were incubated for 10 min at 50 °C. Samples were then incubated with iodoacetamide (9 mm final concentration) for 30 min at room temperature. The protein was digested overnight with trypsin at 37 °C, and the liberated peptides were desalted using Poros R3 beads (Applied Biosystems) with elution in three cycles of 50% v/v acetonitrile. The peptides were dried in a vacuum centrifuge and resuspended in 10 μl of 5% (v/v) acetonitrile, 0.1% (v/v) formic acid solution.

Digested samples were analyzed by LC-MS/MS using an UltiMate® 3000 Rapid Separation LC (Dionex Corporation, Sunnyvale, CA) coupled to either a LTQ Velos Pro or Orbitrap elite (Thermo Fisher Scientific, Waltham, MA) mass spectrometer (28). Peptides were separated using a gradient from 99% A (0.1% (v/v) formic acid in water) and 1% B (0.1% (v/v) formic acid in acetonitrile) to 25% B, in 20 min at 200 nl/min, using a 75 × 250-mm 1.7 μm BEH C18, analytical column (Waters). Peptides were selected for fragmentation automatically by data-dependent analysis. Data produced were searched using Mascot (Matrix Science UK) against an in-house database that contained the OAM sequence. Methionine oxidation and MTSL addition were selected as variable modifications. MTSL modification was specified in UNIMOD with the accession number 911. The data were validated using Scaffold (Proteome Software, Portland, OR) and then manually validated using Xcalibur Qual browser v2.1 (Thermo Fisher Scientific, Waltham, MA).

##### Continuous Wave (CW), Freeze Quench EPR, and PELDOR Measurements

For CW EPR measurements 250 μm holo wild-type OAM or OAM variants (prepared by mixing 250 μm apoenzyme with equimolar amount of AdoCbl and PLP) were mixed with 10 mm DABA and after 5 min of incubation (350-μl final volume), samples in EPR tubes were frozen in liquid nitrogen. Continuous wave EPR spectra were measured at X-band using a Bruker ELEXSYS E500 spectrometer equipped with a Super High Q resonator (Bruker ER-4123-SHQ). Temperature control was provided by an Oxford Instruments ESR900 helium flow cryostat connected to an ITC503 temperature control unit from the same manufacturer. The EPR spectra were recorded at 20 K as reported previously (25).

Anaerobic freeze quench EPR samples were prepared in the dark using a Perspex premixer unit comprising two female Luer inlet ports, a T-mixer, and male Luer exit port. The inlet ports were fitted with two 1-ml syringes that were filled in the anaerobic cabinet with OAM (189 or 393 μm) and 100 mm ornithine, respectively. A 15-cm needle attached to the exit port of the mixing block delivered the mixed sample into the bottom of a nitrogen-filled EPR tube that was capped with a Suba-Seal closure. The syringes were manually driven in tandem to deliver 0.2 ml from each into the EPR tube, which was then frozen rapidly in a dry ice/ethanol bath; this process took a minimum of 2 s.

Pulsed electron electron double resonance (pulsed ELDOR, PELDOR, or DEER) measurements at X-band were made using a Bruker ELEXSYS E580 spectrometer equipped with an ER-4118X-MD5 dielectric resonator. Temperature control was provided by an Oxford Instruments CF935 helium flow cryostat controlled by an ITC503 temperature controller unit. PELDOR measurements employed the four pulse experiment with the detection frequency pulse train π/2-τ-π-τ-T-π-T-acquire to obtain the refocused echo, whereas the π pump pulse applied at the pump frequency was incremented in 8-ns steps through the τ + T period between the two π pulses at time *t* (29). Therefore the modulation of the refocused echo is given by *V*(*t*) = cos(ν_DD_(*t* − τ)), and thus the interelectron dipolar couplings, ν_DD_, were determined using cosine Fourier transform of the baseline corrected four pulse PELDOR data to produce dipolar spectra. The raw PELDOR data were baseline-corrected using a second order polynomial, and then a Hamming window function was applied to increase signal to noise following the subsequent Fourier transform and drive the decay function to completion, thus avoiding truncation effects. Each data set was then zero-filled to 1024 points prior to Fourier transformation. From the values of ν_DD_ determined from the Fourier transformed data, interelectron distances were obtained using the following equation,

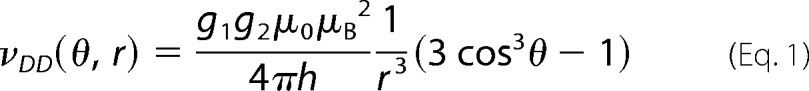
 where *g*_1_ and *g*_2_ are the g values of the two spins, *r* is the distance between them, and θ is the angle between the interspin vector and the applied magnetic field. All EPR samples were analyzed in 4-mm-outer diameter quartz tubes supplied by Wilmad.

## RESULTS AND DISCUSSION

To reconcile the resting state structures obtained for OAM and related proteins with evidence for a short distance Co(II):substrate radical pair formed during catalysis, it was suggested these enzymes couple domain dynamics to radical formation (7, 11–13). Direct evidence for such a dynamic model is lacking, although indirect evidence was obtained during structure determination of OAM following turnover and through modeling using the structural homology with other non-PLP containing cobalamin-dependent enzymes. We used EPR of spin-labeled OAM to probe for the presence of a conformational equilibrium in solution following external aldimine formation.

### 

#### 

##### Pulsed Electron Double Resonance of Double Spin-labeled Resting State OAM

**FIGURE 2.**
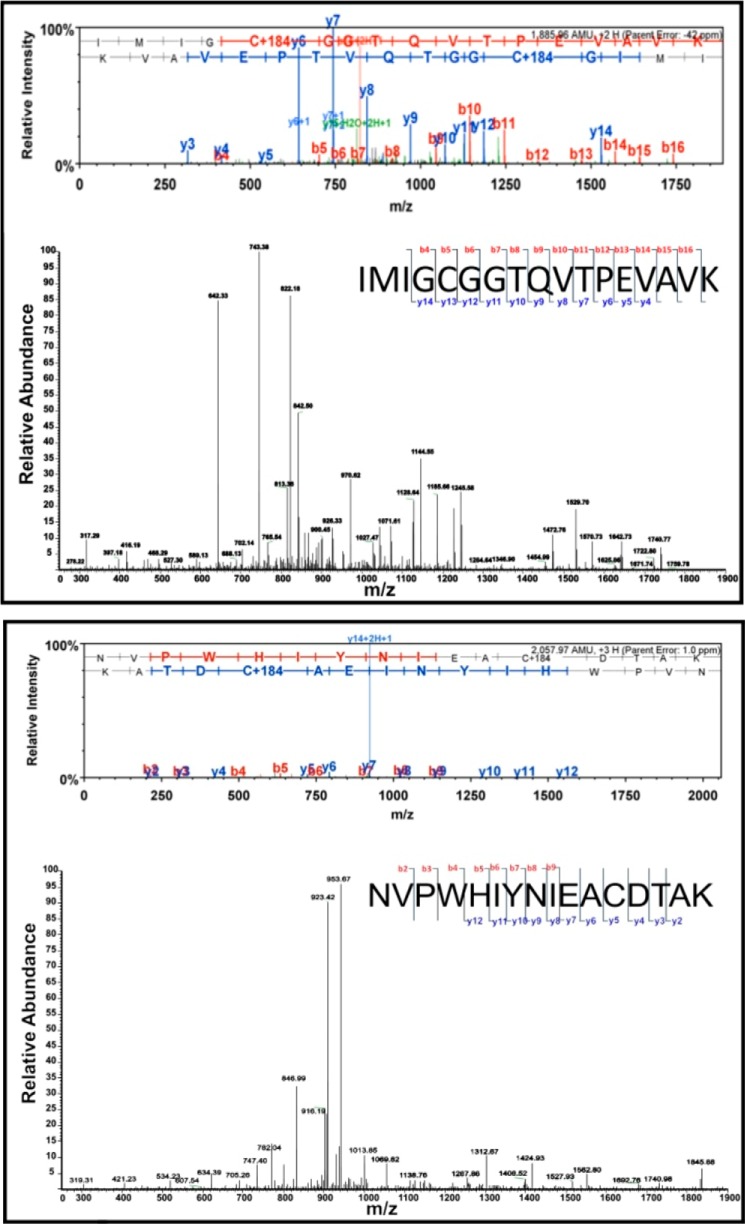
**Mass spectral analysis of MTSL nitroxide spin-labeled wild-type OAM at Cys^700^ and Cys^352^.** The tryptic peptide fragments IMIGCGGTQVTPEVAVK containing Cys^700^ and NVPWHYNIEACDTAK containing Cys^352^ were the precursors selected for the analysis. Peptide fragments are indicated by *b* if the charge is retained on the N terminus and by *y* if the charge is retained on the C terminus. In the standard spectrum format, scaffold shows the *y* ions in *blue* and the *b* ions in *red*. Peaks in *black* have not been associated with standard ions. Scaffold adds the *blue* and *red horizontal ladders* to easily show the identified portion of the amino acid sequence, reading from *left* to *right* for the *red ladder* (*b* ions) and from *right* to *left* for the *blue* (*y* ions). Additionally the MTSL-modified Cys residue has been identified in both *b* and *y* ion series; its mass corresponds to the mass of cysteine plus 184.08 Da.

**FIGURE 3.**
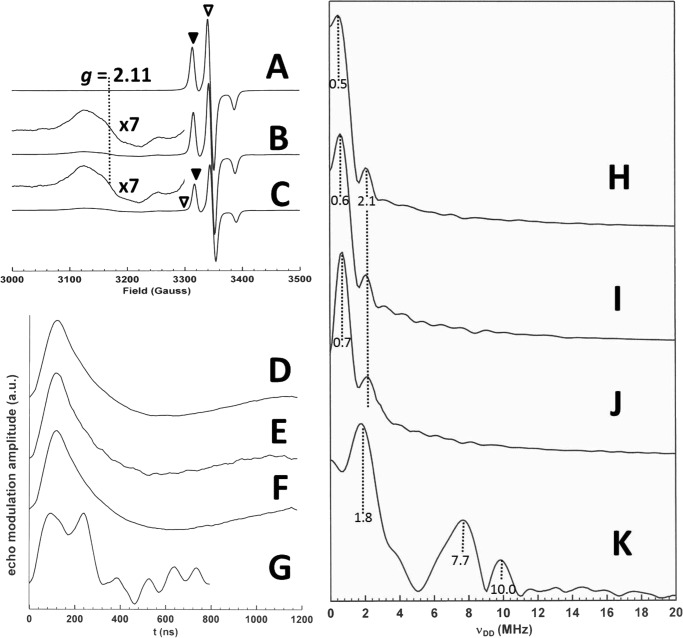
**PELDOR traces obtained for MTSL labeled wild-type OAM and C700S.** *Traces A–C* represent the continuous wave EPR spectra, whereas the PELDOR Fourier transformed data are shown in *traces D–K*. CW EPR spectra of OAM spin-labeled at Cys^352^ and Cys^700^ prepared in the dark (*A*) and OAM spin-labeled at Cys^352^ and Cys^700^ plus DAB (*B*); OAM spin-labeled at Cys^352^ (C700S variant) plus DAB (*C*) are shown. In *B* and *C*, the Co(II) radical coupled signal *g*_⊥_ at *g* = 2.11 is marked. The experimental conditions were as follows: microwave power, 10 μW; modulation amplitude, 5 G; modulation frequency, 100 KHz; and temperature, 80 K. *D* is the time domain PELDOR modulation data, and *H* is its Fourier transform obtained from the sample shown in *A* by observing at ▾ and pumping at ▿ at 80 K; *E* is the time domain PELDOR modulation data, and *I* is its Fourier transform obtained from the sample shown in *B* by observing and pumping as in *A* at 80 K; *F* is the time domain PELDOR modulation data, and *J* is its Fourier transform obtained from the sample shown in *B* by observing and pumping as in *A* at 15 K; *G* is the time domain PELDOR modulation data, and *K* is its Fourier transform obtained from the sample shown in *C* by observing at ▾ and pumping at ▿ at 10 K. The experimental conditions for *D* and *E* were as follows: τ = 200 ns; *T* = 1100 ns; shot repetition time, 3 ms; *t* incremented in 148 8-ns steps; and temperature, 80 K. The experimental conditions for *F* were as follows: τ = 200 ns; *T* = 1100 ns; shot repetition time, 2 ms; *t* incremented in 148 8-ns steps; and temperature, 15 K. The experimental conditions for *G* were as follows: τ = 190 ns; *T* = 732 ns; shot repetition time, 10 ms; *t* incremented in 100 8-ns steps; and temperature, 10 K.

To validate the dynamic model proposed for OAM (Fig. 1), WT OAM was spin-labeled at accessible cysteine residues. LC-MS/MS of a tryptic digest routinely revealed labeling occurs at Cys^352^ and Cys^700^, and near quantitative labeling (>90%) was observed (Fig. 2). The catalytic properties of the labeling enzymes were similar to the unlabeled WT OAM as demonstrated by steady-state measurements (Table 1). OAM spin-labeled at Cys^352^ and Cys^700^ prepared in the dark gave the frozen solution CW EPR spectrum shown in Fig. 3*A*. This spectrum shows only the “triplet” of three lines centered on *g* = 2.00 expected for the MTSL spin label in frozen solution (30). No other signals attributable to low spin cobalamin derived Co(II) are evident. The Fourier transform of the four pulse PELDOR data are presented in Fig. 3*H*, and two values for ν_DD_ are evident, 0.5 and 2.1 MHz with the latter having lower intensity than the former. These ν_DD_ values correspond to distances of 47 and 29 Å, respectively. Examination of the published x-ray crystal structure of OAM in the resting state (Protein Data Bank code 3KP1) reveals that within the OAM dimer the Cys^700^–Cys^700^′ (where ′ indicates the residue on the opposite monomer) and Cys^700^–Cys^352^′ distances are too large to be measured using the PELDOR parameters employed here (11). However, the measurable Cys^700^–Cys^352^ and Cys^352^–Cys^352^′ distances are 49 and 29 Å, respectively, suggesting that these are the distances measured in the PELDOR experiment. The relatively low intensity of the ν_DD_ = 2.1 MHz feature indicates that spin labeling at Cys^352^ may not be complete, leading to a weaker signal for the interaction between spin labels at the Cys^352^ positions in the dimer.

**TABLE 1:** **The steady-state kinetic parameters for wild-type OAM and C700S variant before and after MTSL nitroxide spin labeling** A coupled spectrophotometric assay with DAPDH, where enzyme activity was measured by following an absorbance increase at 340 nm, corresponding to reduction of NAD^+^ to NADH, was used to determine the kinetic parameters. Kinetic assays were performed in a 1.0-ml volume at 25 °C using a 1-cm-path length cuvette. To make a functionally active holoenzyme, equimolar amounts of PLP and AdoCbl were added to the apo form of the protein. The reaction mixtures contained 100 nm holoOAM, 100 nm DAPDH, 0.5 mm NAD^+^, and variable concentrations of d-ornithine (0–2250 μm).

Enzyme	Cysteine residues labeled with MTSL	*k*_cat_	*K_m_*	*k*_cat_/*K_m_*
				× *10^3^m*^−*1*^ *s*^−*1*^
Wild-type OAM unlabeled	NA*^a^*	2.97 ± 0.01	189 ± 14	15.7 ± 1.2
Wild-type OAM MTSL labeled	Cys^352^, Cys^700^	2.86 ± 0.03	186 ± 13	15.4 ± 1.1
C700S unlabeled	NA	2.88 ± 0.03	186 ± 11	15.5 ± 0.9
C700S MTSL labeled	Cys^352^	2.78 ± 0.08	189 ± 6	14.7 ± 0.6

*^a^* NA, not applicable.

##### Pulsed Electron Double Resonance of Double Spin-labeled OAM:DAB

We have shown previously that addition of the inhibitor DAB to OAM gives rise to a coupled Co(II) radical species with a distinctive EPR spectrum having *g*_⊥_ = 2.11 (25). This behavior is duplicated in the doubly spin-labeled preparation, giving the CW EPR spectrum of Fig. 3*B*. Performing a PELDOR experiment on this sample yielded the data of Fig. 3*I*, again indicating two values for ν_DD_: 0.6 and 2.1 MHz; the latter is again assigned to the distance between Cys^352^ and Cys^352^′. The smaller coupling corresponds to a distance of 44 Å and thus a slight shortening of the distance between Cys^700^ and Cys^352^. Both distances remain in keeping with the x-ray crystal structure of the DAB-bound OAM prepared under anaerobic conditions (Protein Data Bank code 3KOY) (11). However, the relaxation behavior of the spin label is surprising. Given the relatively rapid electron spin relaxation exhibited by low spin Co(II) species, that the PELDOR data of Fig. 3*I* can be obtained with the same parameters as that of Fig. 3*H* suggests that the former data arises from that portion of OAM that has not formed the Co(II) radical species. A re-examination of the PELDOR experiment revealed a second species for which ν_DD_ values could be obtained at 15 K, shown in Fig. 3*J*. Here the ν_DD_ values of 0.7 and 2.1 MHz indicate another small decrease in the Cys^700^–Cys^352^ distance to 42 Å. Such a distance between Cys^352^ and Cys^700^ is incompatible with the close approach of substrate and cobalamin necessary for the formation of the Co(II) radical pair, and we propose that this must reflect the conformation of one half of the dimer in which the other half has formed the Co(II) radical pair. This nearby Co(II) species acts to decrease the relaxation time of the spin labels in the other half, thus allowing the PELDOR data to be obtained at 15 K. Unfortunately, PELDOR data from spin label pairs corresponding to the Co(II) population and showing the interlabel distances predicted from model structures of the enzyme conformations that may exist during catalysis (11), and indirectly by the formation of the Co(II) radical pair (25), proved impossible to acquire. PELDOR data obtained from OAM doubly spin-labeled with MTSL at Cys^700^ and Cys^352^ is thus consistent with published x-ray crystal structures representative of the resting state for the enzyme (11). Furthermore, the interlabel distances extracted from OAM:DAB are similar to that obtained for the resting state, despite formation of a Co(II) radical pair in the sample. This suggests that the conformational equilibrium remains poised toward the resting state following external aldimine formation, a conclusion that was also reached during crystallographic studies of OAM. It is important to realize that there are caveats when extending this observation to the behavior of the enzyme at ambient temperatures because both crystallographic and EPR data are collected on samples at cryogenic temperature, which can potentially affect the position of the conformational equilibrium.

##### Pulsed Electron Double Resonance of Single Spin-labeled OAM:DAB

To directly confirm the presence of a distinct conformation linked to Co(II) formation, we used dipolar coupling between a single MTSL label and the Co(II) radical pair. A C700S variant was created, and near quantitative labeling (>90%) was routinely observed at Cys^352^ as verified by LC-MS/MS of a tryptic digest. The catalytic properties of the labeled C700S variant were similar to the unlabeled WT OAM (Table 1). The Co(II) radical pair could be used as one of the two unpaired electron spins in a PELDOR experiment, although this has not previously been achieved for any cobalamin-dependent enzyme. The OAM C700S variant allowed for the production of enzyme spin-labeled only at Cys^352^. Treating this enzyme with DAB produced the frozen solution CW EPR spectrum shown in Fig. 3*C*. The MTSL spectrum and the Co(II) radical spectrum are both evident in Fig. 3*C*, the MTSL spectrum being less intense than in Fig. 3*B* because only Cys^352^ is labeled. The PELDOR data shown in Fig. 3*K* was obtained at 10 K with the observed frequency at the low field component of the MTSL triplet and the pump frequency within the Co(II) radical spectrum 76 MHz downfield of the observed frequency. The ν_DD_ values obtained thus represent distances between the Co(II) radical pair and the spin label at Cys^352^, those distances being 31, 19, and 17 Å. These distances are incompatible with the available OAM crystal structures, which refer to the inactive conformation. In contrast, distances observed can be mapped onto models of the OAM active conformation (11, 12): the longest distance corresponds to the modeled distance between the Co(II) radical center and Cys^352^ in the other half of the dimer (Cys^352^′), whereas the shorter distances are consistent with the Co(II) radical to Cys^352^ distance in the same half of the dimer with the two values, possibly reflecting some conformational heterogeneity (11). This clearly supports the dynamic model proposed for OAM catalysis and furthermore establishes that Co(II) formation is strongly coupled to the active conformation.

##### Selected Mutations at the Cobalamin-PLP Domain Interface Do Not Significantly Affect the OAM Resting State Properties

**FIGURE 4.**
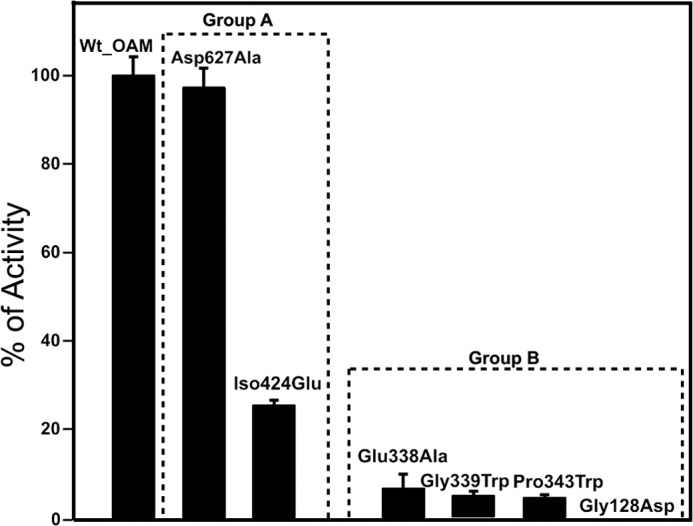
**Catalytic properties of variant enzymes.** Relative activity of the OAM variant enzymes is plotted as the percentage of catalytic turnover number (*k*_cat_) of wild-type OAM. A coupled spectrophotometric assay with DAPDH was used to determine the kinetic parameters for wild-type OAM and variant enzymes.

**FIGURE 5.**
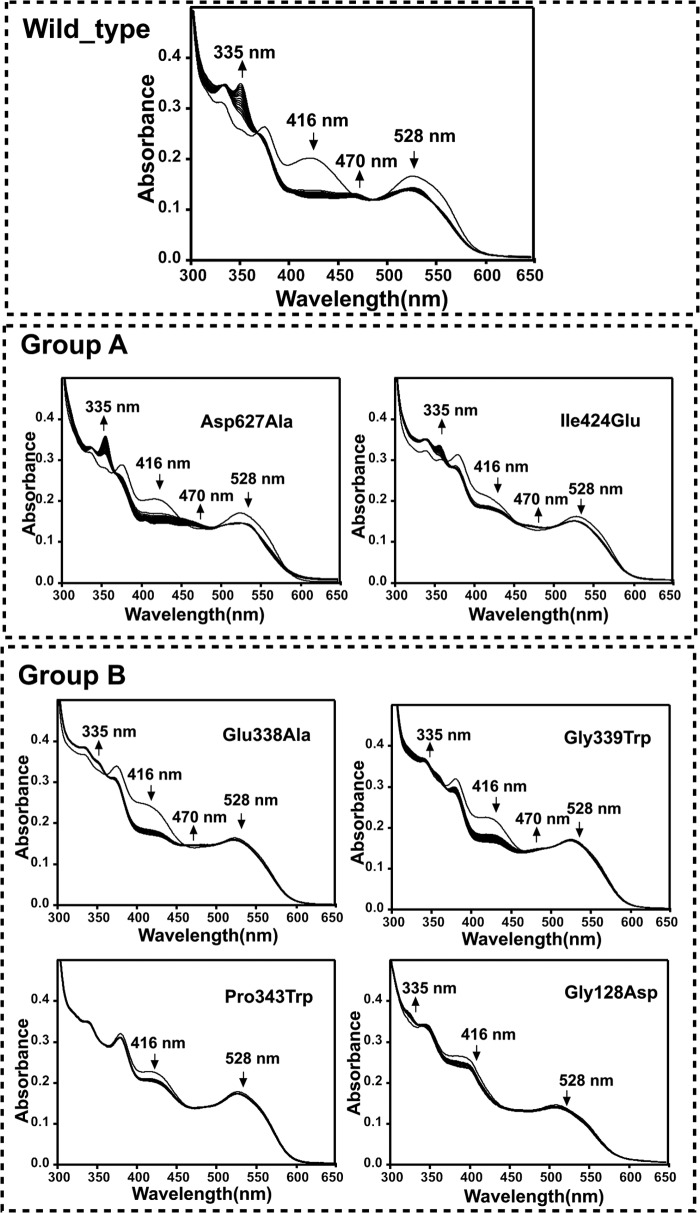
**Change in UV-visible spectra of holo-OAM and variant enzymes induced by binding of the substrate d-ornithine under anaerobic condition.** The holoenzyme solution contained 100 mm NH_4_-EPPS, pH 8.5, 15 μm OAM, 15 μm PLP, and 15 μm AdoCbl in a total volume of 1 ml. Spectral changes for holo-OAM were recorded at 25 °C at 0 and 10 s and then at every 60 s up to 25 min following the addition of 2.5 mm d-ornithine. The *arrows* indicate the direction of absorbance change over time. The absorbance decrease at 528 nm reflects the homolysis of the AdoCbl C–Co bond, the absorbance increase at 470 reflects cob(II)alamin formation, and the decrease in absorbance shoulder at 416 nm corresponds to transimination induced by the d-ornithine binding with PLP.

**FIGURE 6.**
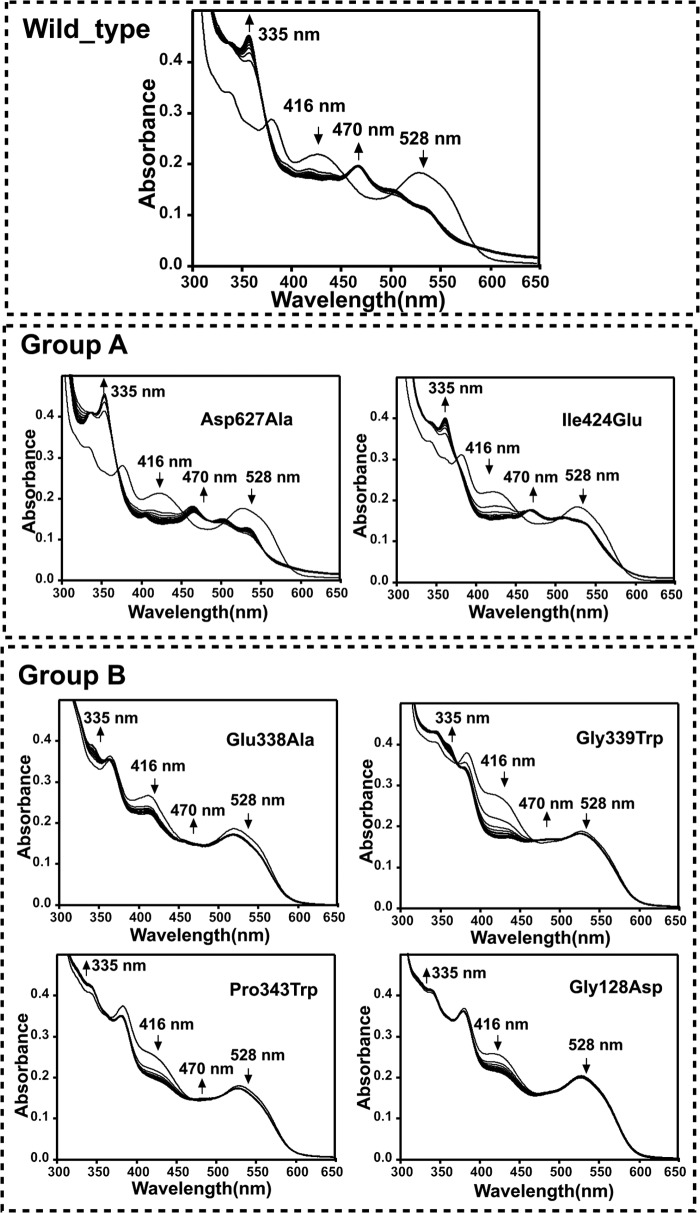
**Change in UV-visible spectra of holo-OAM and variant enzymes induced by binding of the inhibitor DAB under anaerobic condition.** The holoenzyme solution contained 100 mm NH_4_-EPPS, pH 8.5, 15 μm OAM, 15 μm PLP, and 15 μm AdoCbl in a total volume of 1 ml. Spectral changes for holo-OAM were recorded at 25 °C at 0 and 10 s and then at every 60 s up to 25 min following the addition of 2.5 mm dl-2,4-diaminobutryic acid. The *arrows* indicate the direction of absorbance change over time. The absorbance decrease at 528 nm reflects the homolysis of the AdoCbl C–Co bond, the absorbance increase at 470 reflects cob(II)alamin formation, and the decrease in absorbance shoulder at 416 nm corresponds to transimination induced by the DAB binding with PLP.

Unfortunately, the single Co(II)-Cys^352^ MTS distance is not sufficient to fully restrain modeling of the OAM in the Co(II) state and thus validate proposed models. Various models proposed for the active state of the enzyme all share a common feature: the cobalamin domain is docked onto the PLP-binding and substrate-binding TIM barrel in such a manner that the adenosine moiety is in close contact to the PLP-bound substrate. These models also suggest a direct contact between Glu^338^ and the adenosine ribose, supported by mutagenesis studies of Glu^338^ in OAM and corresponding residues in other cobalamin-dependent enzymes (26, 31, 32). To further validate the dynamic model proposed and to delineate the cobalamin domain docking site in the active conformation, we created a range of variants aimed at perturbing the cobalamin domain-PLP-TIM barrel interface. To study this conformational equilibrium in solution, we designed two groups of variants: group A aimed at weakening the interface observed in the resting state crystal structures, and a second group B aimed at selectively weakening the proposed active site interface centered around Glu^338^. Mutations were furthermore limited to those that were unlikely to affect folding/stability or cofactor/substrate binding. Two variants were prepared (group A) that were predicted to affect the cobalamin-PLP domain interface, namely D627A and I424E, respectively, removing a charge interaction and perturbing a hydrophobic interaction as observed in the OAM crystal structures. Four variants (group B) were prepared that perturb the region surrounding Glu^338^. In addition to E338A, mutations G128D, G339W, and P343W were prepared, each introducing a bulky side chain at the proposed interface. The resting state properties of the holoenzyme form of the six OAM variants are similar to those previously established for the WT enzyme (Figs. 4–6).

##### Individual Cobalamin Domain-PLP Domain Interface Mutations Exert Distinct Effects on the Steady-state Properties of the Enzyme

**FIGURE 7.**
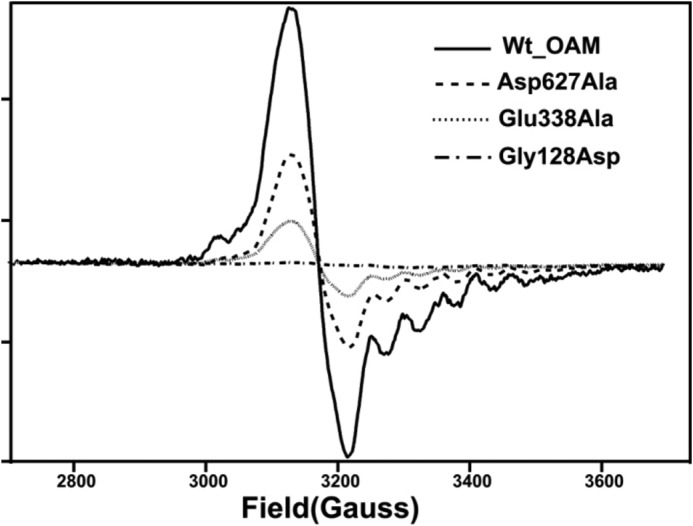
**Continuous wave EPR spectra of wild-type OAM and variant enzymes.** The EPR spectra show the relative amount of paramagnetic species formed for wild-type OAM and variant enzymes in the presence of inhibitor DAB. The holoenzyme solution contained 100 mm NH_4_-EPPS, pH 8.5, 250 μm OAM, 250 μm PLP, and 250 μm AdoCbl. 10 mm dl-2,4-diaminobutryic acid was added to the holoenzyme, and samples were loaded into EPR tubes and after 5 min of incubation time samples were frozen in liquid nitrogen.

**FIGURE 8.**
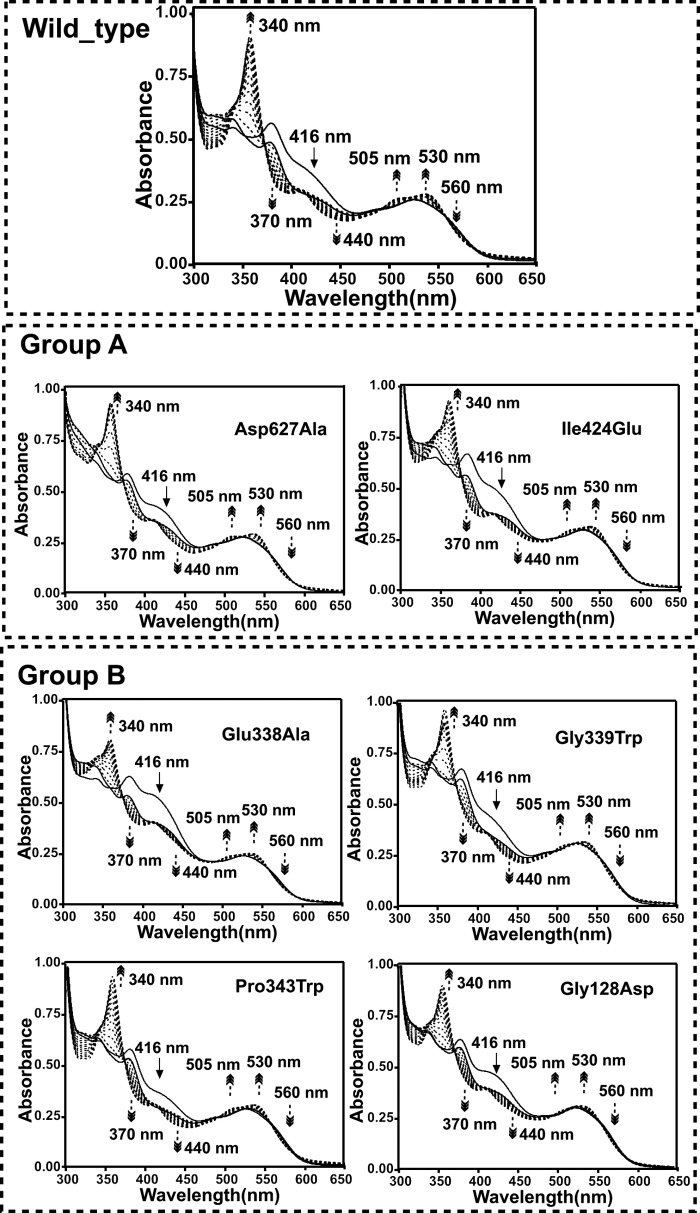
**Change in UV-visible spectra of holo-OAM and variant enzymes bound to inhibitor 3-DAP after induced with a continuous illumination of light under aerobic conditions.** The holoenzyme solution contained 100 mm NH_4_-EPPS, pH 8.5, 30 μm OAM, 30 μm PLP, and 30 μm AdoCbl in a total volume of 1 ml. The UV-visible spectra before and after adding 5 mm 3-DAP are shown as *thick lines*. The *dark arrows* indicate the direction of the absorbance change during inhibitor binding over 25 min. The inhibitor bound holo-OAM was then subjected to continuous illumination from a Schott KL1500 electronic light source after passing the light through a red insert filter (<530-nm cutoff filter), which provides illumination at an intensity of 1000 μmol m^−2^ s^−1^, and spectral changes were recorded at 25 °C at 0 and 10 s and then at every 60 s up to 25 min. The *dark dashed arrows* indicate the direction of the absorbance change during continuous illumination over 25 min.

Despite the lack of any significant difference in the properties of the resting state enzyme, the majority of mutations at the cobalamin-PLP domain interface were found to perturb the steady-steady kinetic properties of the enzyme (Table 2). Although the D627A variant is only mildly affected, all other mutations lead to large reductions in the observed *k*_cat_ values, with little variation in the corresponding *K_m_*. The activity of the G128D mutation was compromised to such a level that steady-state parameters could not be obtained (Fig. 4). UV-visible spectroscopy under anaerobic conditions revealed that all variants displayed similar levels of external aldimine formation between the PLP cofactor and the substrate, but with distinct differences in the level of AdoCbl bond homolysis induced on substrate-binding as evident from the absorbance changes at 528 nm (Fig. 5). A similar situation was obtained when using the inhibitor DAB, which leads to a higher proportion of AdoCbl bond homolysis (Fig. 6). In fact, the level of AdoCbl bond homolysis as indicated by both UV-visible and EPR spectroscopy (Fig. 7) correlates well with the steady-state kinetic parameters obtained for each variant form. In fact, little evidence can be obtained for Co(II) formation in the case of the inactive G128D variant. In contrast, when WT or variants are complexed with the inhibitor 3-DAP (an inhibitor that does not induce AdoCbl bond homolysis but does lead to external aldimine formation), both the rate and extent of light-dependent AdoCbl bond homolysis appear unaffected by the various mutations (Fig. 8). These data clearly establish that both PLP adduct and (light-dependent) Co(II) formation itself are largely unperturbed by the various mutations. It thus appears that the link between external aldimine formation and the ligand-induced AdoCbl bond breakage is weaker in the variant OAMs. In fact, the four mutations that were designed to disrupt the interface established in the active, Co(II) state (group B) are much more affected in both steady-state and ligand-induced Co(II) formation levels than both variants with mutations designed to perturb the resting state conformation.

**TABLE 2:** **The steady-state kinetic parameters for wild-type OAM and variant forms** A coupled spectrophotometric assay with DAPDH was used to determine the kinetic parameters for wild-type OAM and variant enzymes. Kinetic assay and measurements were performed at 25 °C in a 1.0-ml-volume, 1-cm-path length cuvette as described under “Experimental Procedures.”

Classification	Enzyme	*k*_cat_	*K_m_*	*k*_cat_/*K_m_*
				× *10^3^m*^−*1*^ *s*^−*1*^
Wild-type	WT OAM	2.97 ± 0.01	189 ± 14	15.7 ± 1.2
Group A	D627A	2.89 ± 0.01	193 ± 14	15.0 ± 1.8
	I424E	0.76 ± 0.04	185 ± 5	4.12 ± 0.24
Group B	E338A	0.24 ± 0.03	176 ± 8	1.34 ± 0.03
	G339W	0.20 ± 0.05	190 ± 6	1.05 ± 0.03
	P343W	0.14 ± 0.02	193 ± 5	0.73 ± 0.09
	G128D	NA*^a^*	NA	NA

*^a^* NA, not applicable.

##### Pre-steady-state Kinetic Analysis of OAM Variants Reveals Rapid Co(II) Formation

**FIGURE 9.**
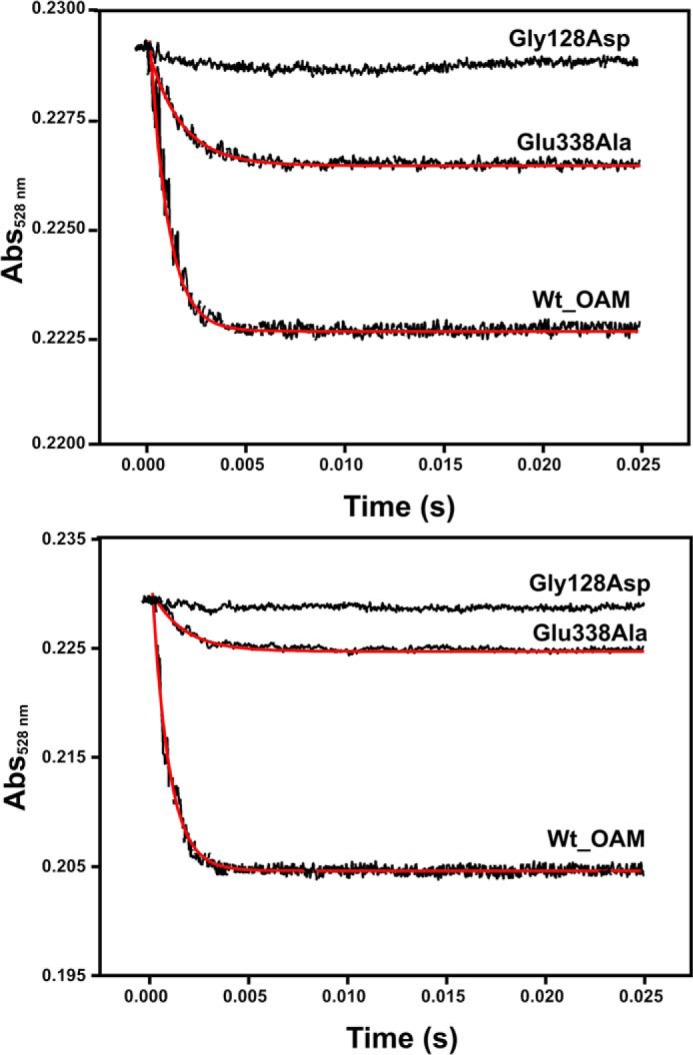
**Anaerobic stopped flow measurement of C–Co bond homolysis in wild-type OAM and OAM variants.** Stopped flow absorbance changes following mixing of holo-OAM and variant enzymes (50 μm; before mixing) with d-ornithine (5 mm; before mixing) and dl-2,4-diaminobutryic acid (5 mm before mixing) under anaerobic conditions at 25 °C. Absorbance change at 528 nm monitors AdoCbl Co-C bond homolysis. An average of 15–20 traces were averaged at each wavelength and used to fit to a single exponential equation to extract the observed rate constants.

Rapid mixing of holoenzyme (both WT and the variant forms) with either substrate (d-ornithine) or inhibitor (DAB) in a stopped flow instrument resulted in rapid absorbance changes at 528 nm, which could be analyzed using a single exponential function (Fig. 9). The observed rate constants were found to be linked to the level of Co(II) formation, with those variants/ligands displaying the highest catalytic activity also having the highest observed rates (Table 3). Unfortunately, because of the very low level of Co(II) formation, no reliable data could be obtained for G128D or P343W with either ligand nor for G339W with d-ornithine. This not only suggests domain motion following external aldimine formation and preceding Co(II) formation remains very fast in the OAM variants but also that the observed rates reflect both forward and reverse rates of the conformational equilibrium established between the resting state and the active Co(II) state. As observed during steady-state measurements, the equilibrium is more perturbed for those variants with mutations that affect the active Co(II) interface (group B). Taken together, these data strongly suggest that Co(II) formation is linked to docking of the cobalamin domain onto the substrate-PLP adduct-bound TIM domain. The position of the equilibrium (but not the rate at which it is achieved) between the active and resting state conformation can be perturbed by mutagenesis of Glu^338^ and surrounding residues on the TIM barrel surface.

**TABLE 3:** **Pre-steady-state measurements and C-Co bond homolysis rate constant for wild-type OAM and variant enzymes** Pre-steady-state kinetic measurements were done in a stopped flow instrument (Applied Photophysics SX.17 MV) in an anaerobic glove box. Absorbance change at 528 nm is monitored up to 0.25 s to follow AdoCbl Co-C bond homolysis rate constant by mixing holo-OAM or holo-OAM variants with d-ornithine or with DABA as described under “Experimental Procedures.”

Classification	Enzyme	DAB	Ornithine
		*s*^−*1*^	*s*^−*1*^
Wild-type	WT OAM	>1000	>1000
Group A	Dp627A	>900	>900
	I424E	877 ± 23	606 ± 19
Group B	E338A	621 ± 154	565 ± 26
	G339W	610 ± 93	NA*^a^*
	P343W	NA	NA
	G128D	NA	NA

*^a^* NA, not applicable.

However, the exact coupling between domain dynamics and radical catalysis could not be established using these data. It is clear that following external aldimine formation, the cobalamin domain rapidly explores the available space to reach equilibrium between the two main populations, the active and resting state. However, whether domain motion is also coupled to radical formation itself is unclear. In the case where domain motion is unrestrained by radical formation, cobalamin domain dynamics following radical formation in the active state could lead to large Co(II) substrate radical distances, with the possible loss of the adenosine moiety or unwanted side reactions at either radical site as a consequence. Alternatively, coupling between radical formation and cobalamin domain dynamics (whereby domain motion can only occur for nonradical states) would require the enzyme to have a mechanism whereby the strength of the cobalamin domain-PLP-TIM barrel interaction is influenced by radical formation.

##### Freeze Quench Trapping of OAM with d-Ornithine Reveals Conformational Heterogeneity

**FIGURE 10.**
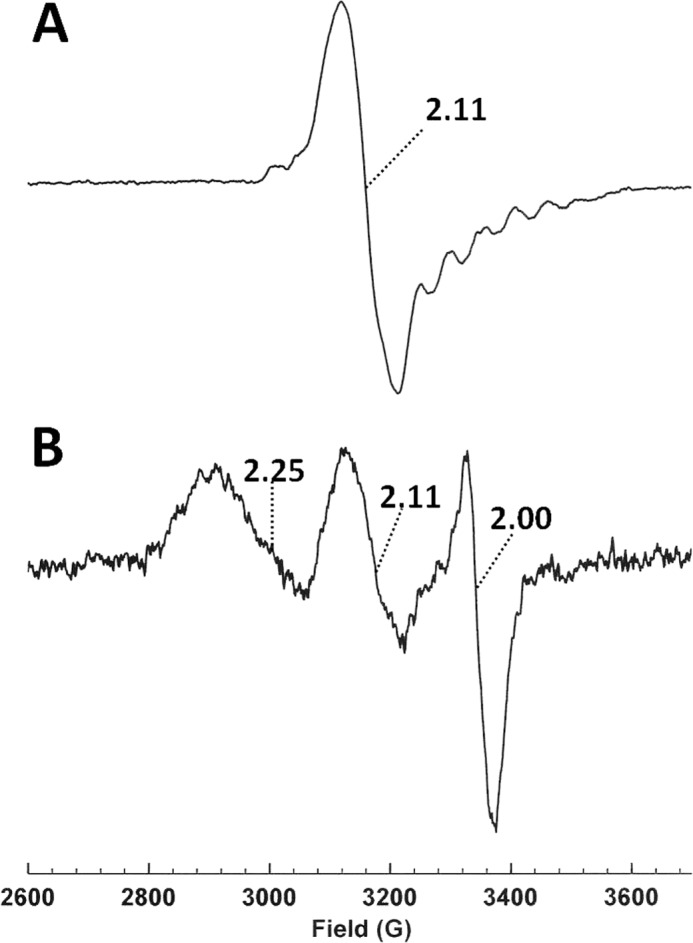
**Continuous wave EPR spectrum of freeze-quenched holo-OAM with d-ornithine.** Continuous wave EPR spectrum of OAM in presence of inhibitor DAB (*A*) and that of holo-OAM d-ornithine rapidly combined with substrate d-ornithine (*B*) using a freeze-quench apparatus is shown. The experimental conditions were as follows: microwave power, 0.5 milliwatt; modulation amplitude, 5 G; modulation frequency, 100 KHz; and temperature, 20 K.

No paramagnetic species was detected in our previous study when OAM turned over the substrate d-ornithine (25). This was attributed to a short half-life for the substrate/product radical states that are rapidly recombined with the abstracted hydrogen atom on 5′-deoxyadenosine to give substrate/product. Therefore a “freeze-quench” technique was employed (see “Experimental Procedures”) in an attempt to trap a paramagnetic species during OAM turnover with d-ornithine. The EPR spectrum subsequently recorded is shown in Fig. 10*B*, with the previously characterized spectrum arising from the reaction of OAM with DAB shown as Fig. 10*A* for reference (25). The three features of Fig. 10*B* can be attributed to the *g*_⊥_ of the Co(II) form of cobalamin at *g* = 2.25, the *g*_⊥_ of a coupled Co(II) radical spin system giving rise to a “hybrid” triplet state, as also observed for the reaction with DAB, at *g* = 2.11 and finally to a “free” radical species at *g* = 2.00 (25). The parallel component counterparts to the two g_⊥_ lines are broad and difficult to detect under the radical signal at *g* = 2.00. The line shapes and *g* values of the EPR spectra that arise from cobalamin-dependent enzyme systems under turnover or with “suicide” inhibitors are dependent on the spin-spin coupling between the unpaired electrons on the Co(II) ion and the radical that constitute the spin system (33). The Co(II) radical signal at *g* = 2.11 can be attributed to enzyme in which the Co(II) to radical distance is 6–7 Å based on analysis of the dipolar contribution to the spin-spin coupling between the two paramagnets (33). The two other signals arise from magnetically isolated, *i.e.* showing no or weak spin-spin coupling, Co(II)balamin and a substrate/product radical that are more than 10 Å apart (33). These data suggest that during turnover with substrate d-ornithine, a conformational equilibrium is established in which the cobalamin-binding domain is able to move relative to the substrate-binding domain. This leads to two apparent Co(II)balamin to substrate/product radical distances being exhibited in the EPR spectrum of OAM with d-ornithine under turnover conditions. EPR spectroscopy of freeze-quench trapped OAM with d-ornithine therefore suggests that domain motion is not coupled to radical formation, because distinct Co(II):substrate radical populations could be distinguished. This mode of action requires a significantly smaller degree of molecular sophistication and would thus appear more robust when compared with a hypothetical mechanism whereby the cobalamin domain motion is severely dampened concomitant with radical formation. It is unclear how loss of the adenosine moiety during domain motion of the cobalamin domain in the Co(II) state is avoided by the enzyme. It is possible domain motion resembles a ball in socket motion, whereby the adenosine is effectively trapped at the ball-socket interface.

##### Conclusion

**FIGURE 11.**
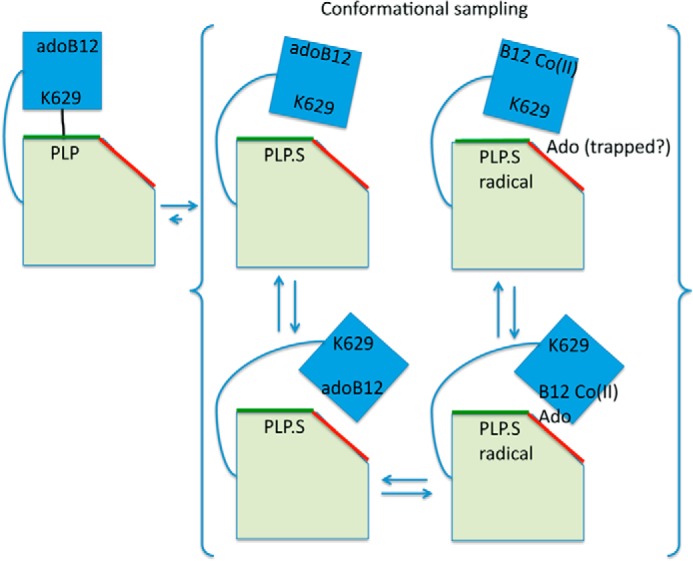
**A dynamic model for OAM catalysis.** Schematic representation of our proposed model for the role of domain dynamics in OAM catalysis. The B12 binding domain is represented by a *blue square*, whereas the PLP binding domain is represented as a *green shape*. The latter has two surfaces indicated by *green* and *red lines*, representing the group A and group B mutations, respectively. Upon substrate binding, the B12 binding domain samples the available space. Our data suggest there is no direct link between radical formation and the B12 domain motion.

Our studies of MTSL-labeled WT OAM and variant OAM enzymes fully support a dynamic model for radical catalysis in the PLP and cobalamin-dependent enzymes. The cobalamin domain motion is tightly coupled to PLP-adduct formation through the release of Lys^629^ from the PLP cofactor. An equilibrium between a resting state conformation and active conformation whereby the cobalamin-adenosine moiety is docked close to PLP-adduct is rapidly established (Fig. 11). The position of this equilibrium can be altered by mutagenesis of the cobalamin domain-PLP domain interface. We could not find evidence for a direct coupling between cobalamin domain motion and radical formation itself. This suggests OAM achieves radical catalysis using a robust design, similar to the dynamic mechanism by which electron-transferring flavoprotein communicates with a wide range of redox partners. In the latter case, largely unguided domain motion of the flavin-containing domain within the confines of the electron transfer complex (*i.e.* conformational sampling) supports rapid electron transfer by transient formation of active complexes.

## Footnotes

3 The abbreviations used are:

OAM: ornithine 4,5-aminomutase
PLP: pyridoxal 5′-phosphate
PELDOR: pulsed electron-electron double resonance
AdoCbl: Adenosyl cobalamin
TIM: triose phosphate isomerase
MTSL: *S*-(2,2,5,5-tetramethyl-2,5-dihydro-^1^H-pyrrol-3-yl)methyl methanesulfonothioate
DABA: dl-2,4-diaminobutyric acid
DAPA: dl-2,3-diaminopropanioc acid
EPR: Electron paramagnetic resonance
DAPDH: (2*R*,4*S*)-2,4-diaminopentanoate dehydrogenase
CW: continuous wave
DAB: 2,4-diaminobutyrate
3-DAP: dl-2,3-diaminopropanoic acid.
